# A Human DPP4-Knockin Mouse’s Susceptibility to Infection by Authentic and Pseudotyped MERS-CoV

**DOI:** 10.3390/v10090448

**Published:** 2018-08-23

**Authors:** Changfa Fan, Xi Wu, Qiang Liu, Qianqian Li, Susu Liu, Jianjun Lu, Yanwei Yang, Yuan Cao, Weijin Huang, Chunnan Liang, Tianlei Ying, Shibo Jiang, Youchun Wang

**Affiliations:** 1 Division of Animal Model Research, Institute for Laboratory Animal Resources, National Institutes for Food and Drug Control, Beijing 100050, China; fancf@nifdc.org.cn (C.F.); wuxi@nifdc.org.cn (X.W.); liususu@nifdc.org.cn (S.L.); caoyuan0512@163.com (Y.C.); chunnan_liang@nifdc.org.cn (C.L.); 2 Division of HIV/AIDS and Sex-Transmitted Virus Vaccines, National Institutes for Food and Drug Control, Beijing 100050, China; liuqiang@nifdc.org.cn (Q.L.); liqianqian1199@163.com (Q.L.); huangweijin@nifdc.org.cn (W.H.); 3 National Center for Safety Evaluation of Drugs, Institute for Food and Drug Safety Evaluation, National Institutes for Food and Drug Control, A8 Hongda Middle Street, Beijing Economic-Technological Development Area, Beijing 100176, China; lujianjun@nifdc.org.cn (J.L.); yangyanwei@nifdc.org.cn (Y.Y.); 4 Key Laboratory of Medical Molecular Virology of the Ministries of Education and Health, Shanghai Medical College, Fudan University, Shanghai 200032, China; tlying@fudan.edu.cn (T.Y.); shibojiang@fudan.edu.cn (S.J.)

**Keywords:** mouse model, hDPP4, MERS-CoV, pseudotyped virus, authentic virus

## Abstract

Infection by the Middle East respiratory syndrome coronavirus (MERS-CoV) causes respiratory illness and has a high mortality rate (~35%). The requirement for the virus to be manipulated in a biosafety level three (BSL-3) facility has impeded development of urgently-needed antiviral agents. Here, we established anovel mouse model by inserting human dipeptidyl peptidase 4 (hDPP4) into the Rosa26 locus using CRISPR/Cas9, resulting in global expression of the transgene in a genetically stable mouse line. The mice were highly susceptible to infection by MERS-CoV clinical strain hCoV-EMC, which induced severe diffuse pulmonary disease in the animals, and could also be infected by an optimized pseudotyped MERS-CoV. Administration of the neutralizing monoclonal antibodies, H111-1 and m336, as well as a fusion inhibitor peptide, HR2P-M2, protected mice from challenge with authentic and pseudotyped MERS-CoV. These results confirmed that the hDPP4-knockin mouse is a novel model for studies of MERS-CoV pathogenesis and anti-MERS-CoV antiviral agents in BSL-3 and BSL-2facilities, respectively.

## 1. Introduction

According to the World Health Organization’s (WHO) latest report, the Middle East respiratory syndrome coronavirus (MERS-CoV) has caused 2121 laboratory-confirmed infections and 740 deaths (~35% mortality rate) in 27 countries since the virus was first isolated in Saudi Arabia in September, 2012 (http://www.who.int/emergencies/mers-cov/en/). Although no cases of the closely-related severe acute respiratory syndrome coronavirus (SARS-CoV) have been reported since 2005 [1], the incidence of MERS-CoV infections and the number of affected countries both continue to increase. Epidemiological trends indicate that prevention of potential epidemic spread of MERS-CoV is likely to be a long and tough battle [2]. Unfortunately, no clinically-approved antiviral monoclonal antibodies (mAbs), small molecule drugs or vaccines against MERS-CoV are available.

Preclinical animal models that mimic the human pathogenesis of MERS-CoV infection are urgently needed for the development of vaccines and therapies. The classification of MERS-CoV as a biosafety level three (BSL-3) agent is an additional hurdle to progress in this area. Since the emergence of MERS-CoV, several animal species have been evaluated as potential models, including wild-type mice, mice deficient in innate immunity [3], Syrian hamsters [4], ferrets [5] and non-human primates (NHPs) [4,6]. Two NHPs (rhesus macaques and marmosets) were found to be susceptible to MERS-CoV infection, but other species including mice are naturally non-permissive to infection. Although human DPP4 (hDPP4) has been identified as the receptor for MERS-CoV [7] that mediates infection, mouse DPP4 (mDPP4) is not functional in this respect [8]. Mice adenovirally transduced with hDPP4 could be transiently infected by MERS-CoV but did not develop fatal MERS disease [9].Moreover, hDPP4-transgenic (Tg) mice expressing hDPP4 under the control of either the cytokeratin 18 promoter [10] or a ubiquitous promoter [11] were susceptible to MERS-CoV infection and developed fatal disease, but also developed unrelated lethal encephalitis. Two knock-in (KI) mice in which mDPP4 was replaced with hDPP4 using CRISPR/Cas9 [8,12] could be infected by high-titer MERS-CoV isolates but were more susceptible to mouse-adapted MERS-CoV strains. Since mDPP4 is central to normal glucose homeostasis [13] and plays an important role in immunity [14], altering its sequence may disrupt these functions.

Neutralizing antiviral mAbs are promising candidates for treatment and prevention of MERS-CoV infection [15]. Several highly potent mouse, human and humanized neutralizing mAbs targeting the receptor-binding domain (RBD) of the spike (S) protein have been reported [16,17,18,19,20], and antiviral compounds and prophylactic vaccines are also under development. These vaccines and therapeutic agents have typically been evaluated in in vitro systems using MERS-CoV pseudoviruses; however, their efficacy must eventually be confirmed in vivo using a suitable animal model. Several Tg or KI mice susceptible to MERS-CoV are available [9,11,12,21], and challenge experiments under BSL-3 conditions have been done for the evaluation of potential therapeutics and vaccines.

In this study, we established a novel KI mouse by inserting the full-length hDPP4 gene into the C57BL/6 mouse genome at the Rosa26 locus (used for constitutive, ubiquitous gene expression) using CRISPR/Cas9 gene editing technology. This mouse, termed R26-hDPP4, displayed severe lung disease related to acute respiratory symptoms (ARDS) as well as central nervous system (CNS) involvement after infection with authentic MERS-CoV clinical isolates at low dose. Moreover, high-titer MERS-CoV pseudovirus could also productively infect R26-hDPP4 mice, with effects comparable to those following authentic infection.

## 2. Materials and Methods

### 2.1. Ethics Statement

Wild-type C57BL/6 mice and genetically-modified mice were supplied by the Institute for Laboratory Animal Resources, National Institute for Food and Drug Control (Beijing, China). All studies were performed in compliance with animal protocols (#2017-B-004) approved by the Institutional Animal Care and Use Committee of the National Institute for Food and Drug Control, China Food and Drug Administration (CFDA, Beijing, China) and in compliance with the “Guide for the Care and Use of Laboratory Animals” (National Academies Press: Washington, DC, USA, 2011; 8th ed.). The license number of the Animal Use Certificate issued by the Science & Technology Department of China (Beijing, China) was SYXK 2016-004, approved on 18 February 2016

### 2.2. Construction of hDPP4-KIMice

To construct the targeting vector, cDNA encoding hDPP4 (also known as CD26 [14]) linked to the red fluorescent protein tdTomato by an internal ribosomal entry site (IRES) sequence was inserted between homologous recombination sequences at the Rosa26 locus. The targeting vector, sgRNA specific for the Rosa26 locus and Cas9 mRNA were microinjected into C57BL/6 zygotes, which were subsequently implanted into pseudo-pregnant mice. Offspring were genotyped using primers Dpp4f (5′-CTGCAGTACCCAAAGACTGTACGGG-3′) and Dpp4r (5′-GACACCTTTCCGGATTCAGCTCACA-3′). The expected amplicon size using these primers was 628bp. Genotype-positive founders were back-crossed with C57BL/6 mice to produce generation F1. Tail tips of four F1-positive mice were subjected to Southern blotting to confirm correct insertion of hDPP4.

### 2.3. Generation of Pseudoviruses

Replication-incompetent HIV virions pseudotyped with MERS-CoVS protein (human beta-coronavirus 2c EMC/2012, AFS88936.1) and expressing firefly luciferase (Fluc) were generated as previously described [22,23]. The MERS-CoV S protein sequence was codon optimized for *Homo sapiens* by GENEWIZ (Suzhou, China) and cloned into an expression vector to yield pcDNA3.1-opti-MERS-CoV-Spike. Briefly, human embryonic kidney (HEK) 293T or 293 cells (both from ATCC) were co-transfected with pcDNA3.1-opti-MERS-CoV-Spike and the lentiviral vector pSG3.Δenv.Fluc [22] using various transfection reagents including Lipofectamine 2000 (Invitrogen, 11668019, Carlsbad, CA, USA), Lipofectamine 3000 (Invitrogen, L3000015, USA), polyethylenimine (Alfa Aesar, 43896, Lancashire, UK), Neofect, VigoFect (Vigorous Biotechnology, T001, Beijing, China), and TurboFect (Thermo Scientific, R0531, Waltham, MA, USA). After incubation for 48 h, culture supernatants were centrifuged at 210× *g* for 5 min, filtered through 0.45-μM filters, and concentrated with 30-kDa ultrafiltration devices (Millipore, Boston, MA, USA). All experiments involving pseudotyped MERS-CoV were performed in a BSL-2facility.

### 2.4. In Vitro Neutralization Tests

To conduct high-throughput in vitro neutralization assays, an optimized dose of MERS-CoV pseudovirus was incubated with each mAb for 1 h at 37 °C, and then the mixture was added to human Huh7 hepatoma cells (Fengh Bio. Inc., Changsha, China) or other cell types in a 96-well plate and incubated for 48 h. The infectivity of MERS-CoV pseudoviruses was determined by measuring bioluminescence as described previously [24].

### 2.5. MERS-CoV-Neutralizing Monoclonal Antibodies

Recombinant extracellular domain of the MERS-CoV spike (S) protein (40069-V08B) from human beta-coronavirus 2c EMC/2012 and its recombinant RBD (40071-V08B1) were purchased from Sino Biological, Inc. (Beijing, China). Phage library construction and antibody selections were carried out as described below. Six-week-old female BALB/c mice were immunized intraperitoneally (I.P.) with 5 µg of recombinant S protein. RNA was extracted from spleen cells and used to construct a phage-displayed antibody library. Recombinant RBD protein was used to screen antibodies. After four rounds of panning, antibodies that specifically recognized the S protein were obtained. The variable regions of the light and heavy chains of these antibodies were fused to mouse kappa or IgG1 constant regions using PCR and cloned into the pSTEP2 HEK-293 transient expression vector. Suspension HEK-293 cells were transfected with light chain and heavy chain expression vectors and recombinant mouse mAbs were purified from culture supernatants using protein A affinity chromatography. The positive control mAbm336 was prepared and provided by Prof. Tianlei Ying [25]. The peptide inhibitor of MERS-CoV was provided by Prof. Shibo Jiang [26,27].

The variable regions of the light and heavy chains of mouse antibody MERS-H111 were aligned to human germline genes, and the most homologous human framework was chosen for humanization [28,29]. The murine sequence was preserved at key residues to maintain structural stability. Codon-optimized light-chain and heavy-chain variable region sequences were synthesized and cloned into expression vectors containing kappa and IgG1 constant regions. Suspension HEK-293 cells were transfected with expression vectors encoding the light and heavy chains of MERS-H111-1, and the expressed antibody was purified from the supernatant using protein A affinity chromatography after 7 days.

### 2.6. Murine Model of MERS-CoV Pseudovirus Infection

Mice were challenged by different routes with varying doses of pseudotyped MERS-CoV. The IVIS-Lumina II imaging system (Xenogen, Baltimore, MD, USA) was used to detect bioluminescence as described previously [30,31]. Prior to measuring luminescence, mice were anesthetized by I.P. injection of sodium pentobarbital (240 mg/kg). The exposure time was 60 s, and fluorescence intensity in regions of interest was analyzed using Living Image software (Caliper Life Sciences, Baltimore, MD, USA). Different wavelengths were used for detecting pseudovirus and tdTomato fluorescence. The substrate, d-luciferin (50 mg/kg, Xenogen-Caliper Corp., Alameda, CA, USA), was injected I.P. and imaging was conducted 10 min later. The relative intensities of emitted light were represented as colors ranging from red (intense) to blue (weak) and quantitatively presented as photon flux in photon/s/cm^2^/sr.

### 2.7. Authentic Virus Infection of Mice and Plaque Assays

Four-week-old R26-hDPP4 and wild-type mice were challenged with authentic MERS-CoV strain hCoV-EMC (1.5 × 10^5^ PFUs) by the intranasal (I.N.) route and body weight was measured daily. On the fifth day post-infection (p.i.), all mice were sacrificed for sample collection. The timing of sacrifice was based on the consideration of humane euthanasia for body weight loss exceeding 25% [8]. Viral titers in lung tissues were determined by plaque assays on Vero cells following a protocol described previously [18]. The authentic MERS-CoV strain hCoV-EMC was maintained and tested in the BSL-3 facility (facility No. ABSL-3059) of the Institute of Laboratory Animal Sciences, CAM & PUMC, Beijing China, with strict use of personal protective equipment as described previously [32].

### 2.8. RNA Extraction and Real-Time QuantitativePCR

Tissues were dissected, immediately immersed in RNAlater^®^ stabilization reagent (Invitrogen, USA) and stored at −80 °C. Total RNA was extracted from individual tissues using TRIzol and quantified using a spectrophotometer at a wavelength of 260 nm. Random hexamers were used to prime reverse-transcription reactions using a reverse transcription (RT)-PCR kit containing SYBR green dye (Takara, Shiga, Japan). Real-time quantitative PCR (RT-qPCR) was performed using a Light Cycler 480 Real-Time PCR system (Roche, Indianapolis, IN, USA). Each reaction was performed in triplicate. Relative expression level of DPP4 was determined using primers DPP4-Q-F (5′-GGGTCACATGGTCACCAGTG-3′) and DPP4-Q-R (5′-TCTGTGTCGTTAAATTGGGCATA-3′) and normalized to expression of GAPDH (glyceraldehyde 3-phosphate dehydrogenase). Viral loads were quantified using primers gag-F1 (5′-AGCACAGCAAGCAGCAGC-3′), gag-R1 (5′-GTGGCTCCTTCTGATAATGCTGAA-3′) and a TaqMan probe (5′Fam-ACAGGAAACAGCAGCCAGGTCAGCCGA-3′Tamra). Data were presented as log viral RNA copies/GAPDH copies.

### 2.9. Western Blotting

Mouse tissues were homogenized in RIPA lysis buffer (50 mM Tris-HCl, pH 7.4, containing 150 mM NaCl, 100 mM EDTA, and 0.1% SDS) supplemented with 1× Proteinase Inhibitor (PI) (Roche, Basel, Switzerland). The denatured protein lysates were separated using 10% SDS-PAGE gels. After transfer, anti-hDPP4 mouse monoclonal antibody (1:3000 dilution, Origene, Rockville, MD, USA) and anti-GAPDH antibody (1:5000, Abcam, Cambridge, UK) were added, followed by horseradish peroxidase (HRP)-conjugated anti-mouse IgG (1:20,000, Santa Cruz Biotechnology, Santa Cruz, CA, USA). The Immobilon Western Chemiluminescent HRP Substrate kit (Millipore Corporation, Billerica, MA, USA) was used for development.

### 2.10. Immunohistochemistry

Mouse tissues were fixed in 10% neutral-buffered formalin, embedded in paraffin, and sectioned to about 3-μm thickness. Tissue sections were stained with hematoxylin and eosin for histopathological examination. For immunohistochemistry (IHC), tissue sections were rehydrated and incubated in Coplin jars filled with Citra buffer (pH 6.0) at 96 °C in a microwave oven for 10 min, cooled at room temperature for 60 min and then blocked with 10% normal goat serum at 37 °C for 60 min. The sections were incubated overnight at 4 °C with either 1:200 rabbit R723mAb (produced by phage display, with specificity against the receptor-binding domain of MERS-CoV; kindly provided by Beijing Wantai Biological Pharmacy Enterprise Co., Ltd., Beijing, China) or normal goat serum (control). Sections were washed with PBS and incubated with HRP-conjugated goat-anti-rabbit secondary antibody (Zhongshan Golden Bridge Biocompany, Beijing, China) for 40 min at room temperature, followed by development with 3,3′-diaminobenzidinesubstrate (Zhongshan Golden Bridge Biocompany, Beijing, China) and counterstaining with hematoxylin.

### 2.11. Data and Statistical Analysis

Sample sizes in each group were calculated based on anticipated effect sizes to yield statistically significant differences. Inhibition rates of mAbs were calculated using the following formula: (value of positive group−value of mAb treatment group)/value of positive group. The value represented either fluorescence derived from pseudovirus infection or authentic viral titers in lung tissues. Data were analyzed using SPSS (ver. 18.0; IBM, Armonk, NY, USA) or GraphPad Prism 5.0 (GraphPad Software, San Diego, CA, USA). Results from each experiment were presented as means plus standard deviations (SD) or standard error of mean (SEM). Student’s *t*-tests were used to assess differences between groups. Dunnett’s tests were performed for multiple comparisons. *p* values less than 0.05 were considered statistically significant.

## 3. Results

### 3.1. A HDPP4-Knockin Mouse (R26-hDPP4) Was Established Using CRISPR/Cas9

**Figure 1:**
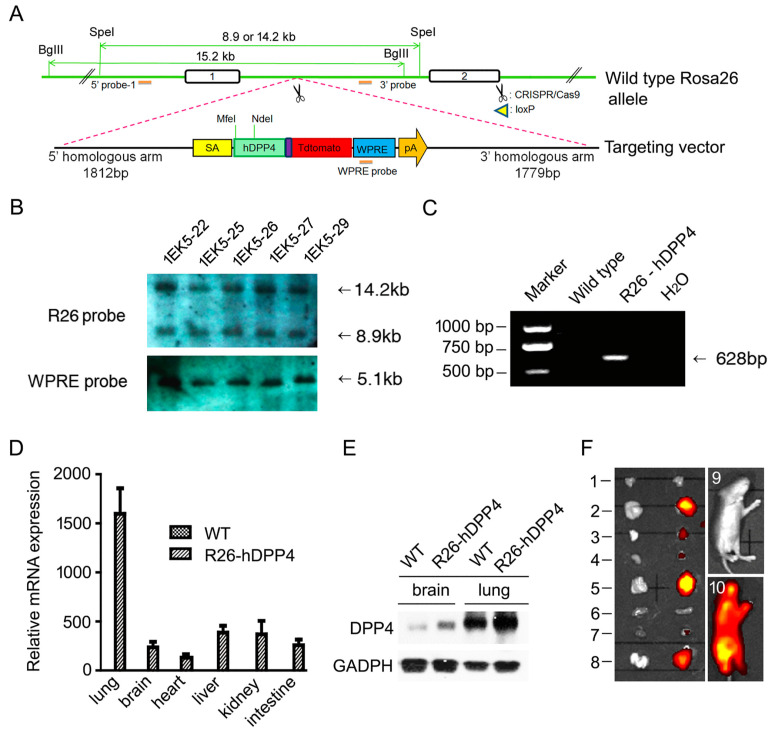
Establishment of a R26-hDPP4-knockin mouse model. (**A**) Schematic strategy for generation of R26-hDPP4-knockin mice via CRISPR/Cas9. (**B**) For the R26 probe, genomic DNA was digested with *Spe*I, and the expected sizes of wild-type and gene-targeted bands were 8.9 kb and 14.2 kb, respectively. For the WPRE probe, genomic DNA was digested with *Bgl*II, and the expected size of the gene-targeted band was 5.1 kb. Four representative results are shown. (**C**) PCR genotyping of R26-hDPP4 mice. The primer pair was designed to anneal in the coding region of hDPP4, and the expected PCR amplicon was 628 bp in length. (**D**) Quantitative reverse transcription PCR (RT-qPCR) of *hDPP4* mRNA in R26-hDPP4 mice and wild-type C57BL/6 mice. Values are presented as means ± SEMs of three independent experiments and were normalized to GAPDH levels. No expression of R26-hDPP4 in wild-type mice was detected. (**E**) Detection of DPP4 protein by western blotting in brain and lung. Weaker blotting signal was detected in wild-type mice, indicating the anti-hDPP4 antibody could recognize mDPP4. (**F**) Bioluminescence imaging (BLI) of newborn R26-hDPP4-knockin mice showing hDPP4 expression in their organs. The imaged organs were: (1) thymus, (2) liver, (3) stomach, (4) kidney, (5) intestine, (6) spleen, (7) heart, (8) lung, (9) and (10) the whole bodies of wild-type mice and R26-hDPP4 mice, respectively.

A KI mouse was generated by inserting the full-length hDPP4 gene [7] into the Rosa26 locus, which has been shown to be a safe harbor for developing genetically stable KI mouse lines [33]. Expression of hDDP4cDNA was driven by the splice acceptor, which allowed ubiquitous expression of hDPP4 under control of the Rosa26 promoter. The tdTomato gene was inserted downstream of hDPP4 with an internal ribosome entry site allowing co-expression of tdTomato and hDPP4. A poly(A) sequence and a woodchuck hepatitis virus posttranscriptional regulatory element (WPRE) were added to enhance mRNA stability and translation efficiency (Figure 1A). The targeting vector along with subgenomic RNA (sgRNA) and Cas9 mRNA were injected into zygotes of C57BL/6 mice. Successful insertion in nine of 41 offspring was positively confirmed by Southern blotting and the results for five mice are presented in Figure 1B. One male founder and one female founder died before weaning and two founders were too emaciated to mate. Five founders were backcrossed with C57BL/6 mice, and two matings produced F1-positive offspring. Tail tips of F1-positive mice were subjected to PCR genotyping (Figure 1C) and Southern blotting to confirm correct insertion of hDPP4. Homozygous mice were termed B6-Gt (Rosa) 26 Sortm1 (SA-hDPP4-tdTomato), abbreviated to R26-hDPP4. Significant hDPP4 expression could be detected by RT-PCR in all R26-hDPP4 mouse tissues tested (Figure 1D), but no expression was detected in wild-type mice. Western blotting of lung and brain tissues confirmed hDPP4 expression (Figure 1E). Unlike Tg mice, which showed the highest-level expression of hDPP4 in heart and brain [11], R26-hDPP4 mice had the highest hDPP4 expression in lung. Profiting from the insertion of tdTomato downstream of hDPP4, the global expression patterns of hDPP4 were examined visually (Figure 1F) via bioluminescent imaging (BLI). Since the liver, intestine and lung have bigger volumes, they have brighter images than other organs.

### 3.2. R26-hDPP4 Mice Were Susceptible to Authentic MERS-CoV Infection, with Infected Mice Exhibiting Disease Symptoms Similar to Those of MERS-CoV-Infected Human Patients

**Figure 2:**
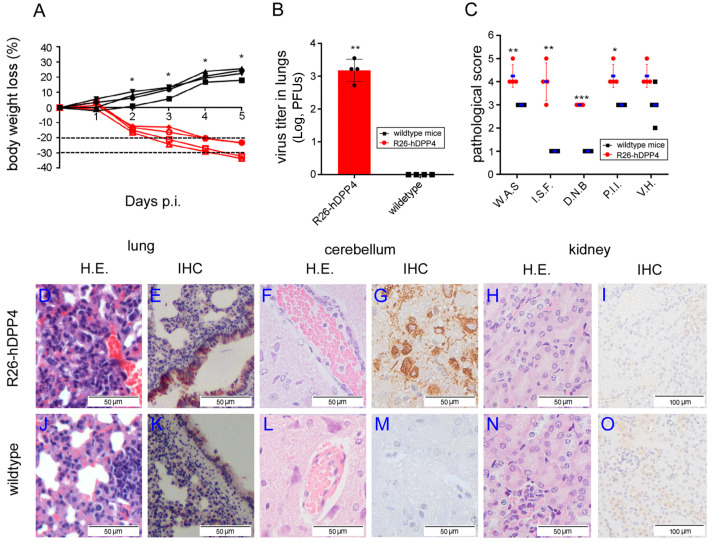
R26-hDPP4-knockin mice were susceptible to infection by authentic MERS-CoV at low dose. (**A**) Weight loss in R26-hDPP4 mice challenged with hCoV-EMC at a dose of 1.5 × 10^5^ PFUs. (**B**) Viral titers in lungs of challenged R26-hDPP4 mice on day 5 p.i. LOD (limitation of detection): 0.85 PFU. (**C**) Quantitative analysis of pathological lesions in lungs. W. A. S. = widened alveolar septa; I. S. F. = inflammatory cells, serous and fibrinous exudation; D. N. B. = degeneration and necrosis of bronchial epithelial cells; P. I. I. = perivascular inflammatory cell infiltration; V. H. = vasodilator hyperemia. (**D**–**O**) Histopathological changes and viral loads in the lungs, brains, and kidneys of mice. R26-hDDP4 mice exhibited disease symptoms similar to those of MERS-CoV-infected human patients (**D**), while no or mild symptoms were observed in wild-type mice. (**J**). Perivascular gliosis in the cerebellum was observed in R26-hDPP4 mice (**F**) but not in wild-type mice (**L**). No pathological lesions were identified in the kidneys of either R26-hDPP4 or wild-type mice (**I**,**O**). IHC assays confirmed viral loads in lungs (**E**) and cerebella (**G**) of R26-hDPP4 mice; little or no virus was detected in the lungs (**K**) and cerebella (**M**) of wild-type mice. Four mice in each group were infected, and samples from all mice were subjected to tittering and histopathological analysis (* *p* < 0.05; ** *p* < 0.01; *** *p* < 0.001).

To test whether R26-hDPP4 mice could support efficient infection by and replication of MERS-CoV, 4- to 5-week-old R26-hDPP4 and wild-type mice were infected I.N. with MERS-CoV clinical strain hCoV-EMC [34] at a dose of 1.5 × 10^5^ PFUs. The bodyweights of wild-type mice increased approximately 24% (Figure 2A) by day 5 p.i., while R26-hDPP4 mice experienced significant weight loss of up to 28% (23–33.5%). By day 4 p.i., all R26-hDPP4 mice had lost at least 20% of their bodyweights, meeting the typical cut-off used in humane euthanasia criteria [8]. Two mice (2/4) had lost more than 30% of their body weights by day 4 p.i.

Viral titers in the lungs of R26-hDPP4 mice were measured as approximately 10^3^ PFU sat 5 days p.i. while no virus was detected in the lungs of wild-type mice (Figure 2B; mean difference 2.958 log PFUs, 95% CI 1.266–4.699 log PFUs). Although weight loss and viral loads provide important measures of mouse susceptibility, these parameters do not directly reflect pathologic changes. Therefore, the lungs, brains, livers, and kidneys collected from infected mice were subjected to histopathological analysis and IHC (Figure 2C–O). By quantitative analysis of pathological scores, the symptoms of R26-hDPP4 mice were significantly different compared with wild-type mice (Figure 2C). Associated with these pathological changes, significant viral loads were detected in lung (Figure 2E). In the cerebellum, cerebral ganglia and cerebrum of R26-hDPP4 mice, perivascular gliosis was observed (Figure 2F; Supplementary Figure S1C) with accompanying virus detected in this organ (Figure 2G). No obvious lesions were observed in liver and kidney (Figure 2H) and no virus was detected in these organs (Figure 2I), despite the fact that a MERS-CoV-infected patient with acute nephritis has been reported [35]. By contrast, in the corresponding organs of wild-type mice, no or minimal pathological changes were observed and no virus could be detected (Figure 2J–O). These results indicated that R26-hDPP4 mice were permissive to infection by authentic MERS-CoV and that infection was accompanied by severe disease symptoms similar to those of MERS-CoV-infected human patients.

### 3.3. A MERS-CoV S-RBD-Specific Neutralizing Antibody Protected R26-hDPP4 Mice from Challenge with Authentic MERS-CoV

**Figure 3:**
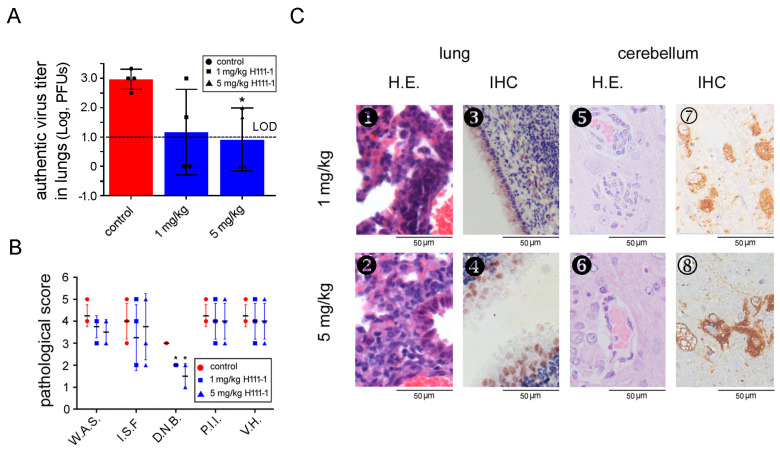
MERS-CoV S-RBD-specific humanized neutralizing antibody H111-1 protected R26-hDPP4 mice from challenge with authentic MERS-CoV. Four-week-old mice were administered either PBS (control), 1 mg/kg mAb H111-1 or 5 mg/kg mAb H111-1 via the I.P. route, and 6 h later they were challenged I.N. with hCoV-EMC (1.5 × 10^5^ PFUs). On day 5 p.i., mice were sacrificed for virus titering and pathological analysis. (**A**) Treatment with mAb H111-1 significantly decreased viral titers in lungs. The dashed line indicates the LOD. (**B**) Efficacy of mAbH111-1 in abating pathological lesions caused by infection with authentic MERS-CoV. Explanation of pathological changes is given in Figure 2. (**C**➊–**C**➇) Histopathological changes and viral loads in lungs and brains of R26-hDPP4 mice administered 1 mg/kg mAb H111-1 (**C**➊,**C**➌,**C**➎,**C**➆) or 5 mg/kg mAb H111-1 (**C**➋,**C**➍,**C**➏,**C**➇), * *p* < 0.05.

Next, we evaluated the effects of a neutralizing mAbon R26-hDPP4 mice infected with authentic virus. To this end, a humanized mAb, H111-1, was generated by phage display methods and administered intravenously (I.V.) ata single dose (either 1 mg/kg or 5 mg/kg) 6 h after infection with hCoV-EMC (1.5 × 10^5^ PFUs). Administration of H111-1 significantly decreased viral titers in lungs (from 3 log PFUs to 0.9 log PFUs and1.2 log PFUS for the 5 mg/kg and 1 mg/kg groups, respectively; Figure 3A), and reduced weight loss in a dose-dependent manner (Table 1). Viral titers in the higher mAb dose group were below the limit of detection. The average inhibition rates of authentic virus were 70% for 5 mg/kg mAb and 60% for 1 mg/kg mAb, respectively. Notably, mAbH111-1 completely ablated viral loads in the lungs in two of four mice in both low- and high- dose mAb groups (Figure 3A). IHC results confirmed lower viral loads in the lungs of mAb-treated mice (Figure 3(C➌,C➍)), while a reduction of viral load in cerebellum was less clear (Figure 3(C➆,C➇)). In addition, treatment with mAb H111-1 alleviated symptoms in both lung and brain (Figure 3(C➊,C➋,C➎,C➏)), with degeneration and necrosis of bronchial epithelial cells improving significantly in treated animals (Figure 3B, *p* < 0.05). These results indicated that humanized mAb H111 is a promising antiviral agent for preventing MERS-CoV infection. Importantly, these results also implied that the R26-hDPP4 mouse could be an effective model for evaluating antiviral agents against MERS-CoV in vivo.

### 3.4. AMurine Model of Infection with Pseudotyped MERS-CoV was Established Using R26-hDPP4 Mice

#### 3.4.1. Optimization of Pseudotyped MERS-CoV System and Establishment of a Model of Pseudotyped MERS-CoV Infection Using R26-hDPP4 Mice

Zhao et al [2] reported a MERS-CoV pseudovirus that allowed for single-round infection of several cell lines expressing hDPP4. However, despite using several proven methods [22], we at first failed to infect R26-hDPP4 mice, which were otherwise permissive to infection by authentic virus, using pseudotyped virus. At first, we attributed this failure to low pseudovirus titer and therefore we optimized the parameters of pseudovirus production (Supplementary Figure S2A–D). Finally, pseudovirus with titers up to 1.27 × 10^7.5^ TCID_50_/mL (Supplementary Figure S2E) was obtained. The pseudovirus was then titrated in 4-week-old R26-hDPP4 mice, and the 50% animal infectious dose (AID_50_) was determined to be 10 TCID_50_/mL viatheintrathoracic (I.T.) route.

To establish an in vitro neutralization assay, the cellular tropism of pHIV/MERSS/Fluc was tested in various cell lines. All 12 cell lines tested were permissive to pHIV/MERSS/Fluc infection, but Huh7 cells showed the highest infection efficiency (Supplementary Figure S2F), demonstrating the wide cellular tropism of the pseudotyped virions. As a result, the Huh7 cell line was chosen to establish a chemiluminescence-based, high-throughput antibody neutralization assay. The assay displayed good sensitivity and specificity.

**Figure 4:**
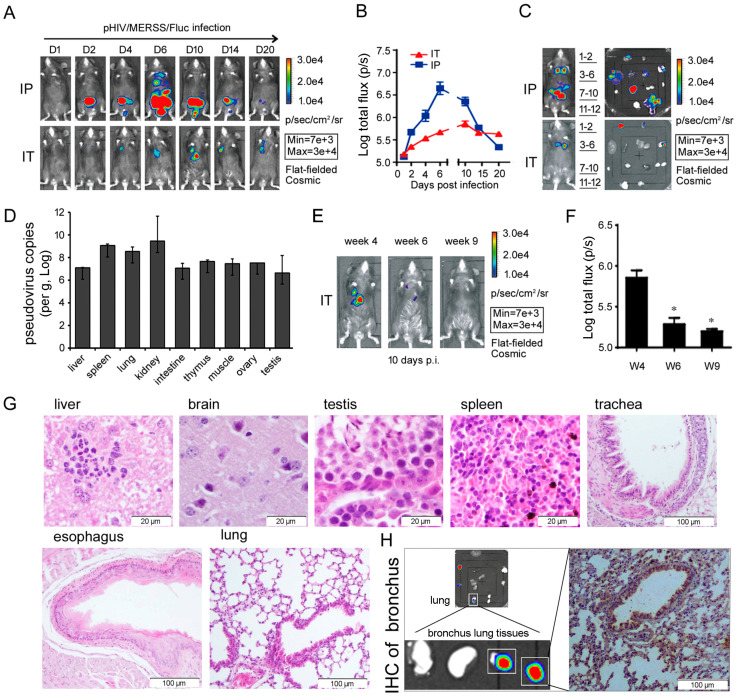
Establishment of the R26-hDDP4 knockin model of infection with MERS-CoV pseudovirus. (**A**–**C**) Four-week-old R26-hDPP4 mice were inoculated with 1.27 × 10^7.5^ TCID_50_ (I.P. route) or 3.8 × 10^6.5^ TCID_50_ (I.T. route) of pHIV/MERSS/Fluc per animal. (**B**) Bioluminescent images (BLI) are shown at different days p.i. Relative bioluminescence intensity was shown in pseudocolor, with red representing the strongest and blue representing the weakest photon fluxes. Data are shown as means ± deviation (**C**). Organs were examined for Fluc expression using BLI: 1 = thymus; 2 = heart; 3 = liver; 4 = spleen; 5 = kidney; 6 = lung; 7 = lymph node; 8 = muscle; 9 = skin; 10 = ovary or testis; 11 = brain; 12 = intestine. (**D**) The copy number of pHIV/MERSS/Flucmeasured by RT-qPCR (I.P. challenge route). (**E**,**F**) Susceptibility tests for mice at different ages. Four- to 9- week-old mice could be infected, but younger mice were more susceptible, * *p* < 0.05. (**G**) Histopathological examination of organs of pseudovirus-infected mice; no histopathological changes were observed. (**H**) Pseudotypedvirions mainly infected the bronchi as shown by IHC and BLI. Bright spots indicate bronchi separated from lung (*n* = 4–6/group).

Since the I.P. infection route was efficient for a pseudotyped Ebola virus model [36], groups of 4-week-old R26-hDPP4 mice were administered pseudotyped virus I.P. Mice were anaesthetized and observed with a BLI system at various days p.i. Bioluminescence of the transgene-encoded Fluc reporter was first observed at day 2 p.i. in the abdomen, close to the injection site (Figure 4A). The bioluminescence then spread to the thoracic cavity by day 6 p.i. Bioluminescence reached peak intensity and then gradually weakened up to day 20 p.i. (Figure 4B). To identify the sites of infection, mice were dissected and their organs were observed on the day of peak infection. Figure 4C showed that thymus, liver, spleen, kidney, lung and muscle, among other organs and tissues, were infected by pseudovirus. Higher pseudoviruscopy numbers were detected in these organs (Figure 4D), illustrating that R26-hDPP4 mice could be efficiently infected by pseudovirus. Since MERS-CoV infection mainly causes clinical ARDS [37,38], the primary site of infection should be the respiratory tract. Therefore, to maximize infection in the respiratory tract, we infected mice via the I.T. route. Live-animal imaging showed that infection was mainly localized to the chest, with maximal infection occurring on days 10 and 11 p.i. Using the I.T. route, the major infected organs were the thymus, heart and lung (Figure 3C). A uniform infection profile was observed in all mice, indicating that I.T. challenge had some advantages over I.P. challenge. I.N. and I.V. challenge were also attempted but did not result in productive infection. To test the influence of the animals’ age on susceptibility to infection, 4- to 9-week-old mice were inoculated with pseudovirus. Both younger and older mice could be infected, but younger mice were more susceptible (Figure 4E,F). As reported previously [30], fluorescence intensity and virus copy number in tissues were strongly correlated, suggesting that fluorescence intensity could be used as an indicator of pseudovirus infection. Pseudovirus infection caused no histopathological changes, indicating that this tool was safe in mice (Figure 4G). Notably, through BLI visualization and confirmation by IHC, we found that pseudovirus tended to infect bronchi (Figure 4H), a tropism shared with authentic virus (Figure 2E).

#### 3.4.2. Relevance of the R26-hDPP4 Mouse Model of Infection by Pseudotyped and Authentic MERS-CoV

**Figure 5:**
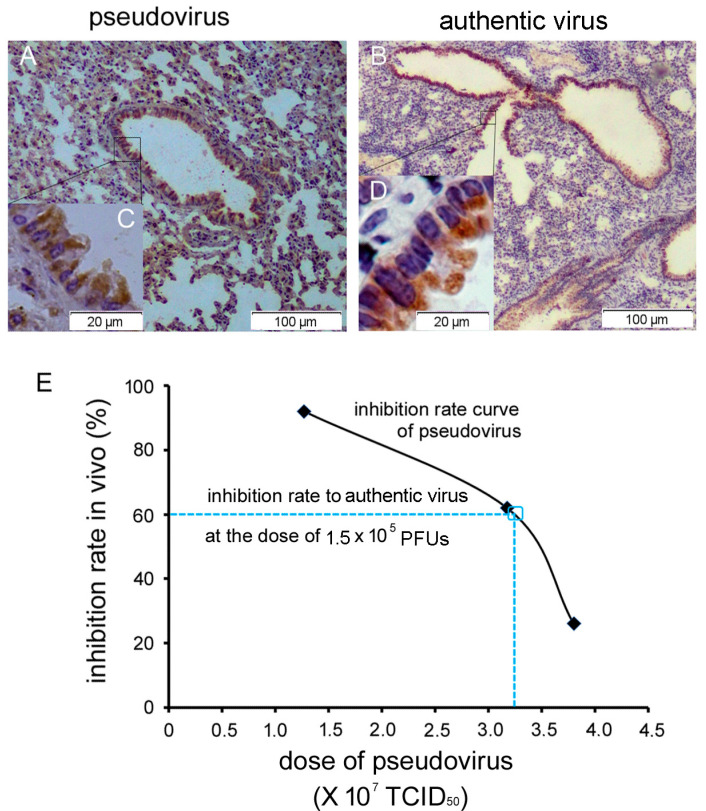
Relationship between pseudovirus and authentic virus models. (**A**–**D**) Pseudovirus and authentic virus infection in the lungs of R26-hDPP4 mice showed a similar pattern, as shown by IHC using mAb R723 against the RBD of the MERS-CoV S protein. (**C**,**D**) Both pseudovirus and authentic virus infected ciliated columnar epithelium of bronchi. (**E**) Dose conversion of pseudovirus and authentic virus. The full black line represented the inhibition rate (◆) of humanized mAb H111-1 (1 mg/kg) in vivo using different pseudovirus doses. The blue dashed line represents inhibition rate of H111-1 against authentic virus. When the dose of authentic virus was 1.5 × 10^5^ PFUs, we calculated that the inhibition rate of H111-1 at a dose of 1 mg/kg would be 60% (see main text). From the inhibition rate curve of pseudovirus, the corresponding pseudovirus dose was 3.25 × 10^7^ TCID_50_. That is, 1 TCID_50_ of pseudovirus corresponded to 0.0046 PFU of authentic virus (1.5 × 10^5^/3.25 × 10^7^). *n* = 4–6 mice per group.

We compared the infection profile between R26-hDDP4 mice infected with pseudotyped and authentic MERS-CoV (Table S1). Lungs were the major infected organs for both types of virus (Figure 5A,B), and pulmonary bronchial epithelial cells were the common sites of infection (Figure 5C,D). Lung tissue tropism was also supported by measurement of pseudovirus copy number (Figure 4D) and authentic virus titering (Figure 2B). The dose conversion of pseudovirus and authentic virus is shown in Figure 5E. Authentic virus was observed in the CNS (Figure 2G and Figure 3(C➆,C➇)) of infected mice. Interestingly, we observed that pseudovirus could also enter the CNS, as it was detectable by qRT-PCR or BLI in the brain. How pseudovirus crosses the blood-brain barrier requires further investigation.

### 3.5. MERS-CoV S-RBD-Specific Neutralizing Antibodies Protected R26-hDPP4 Mice against Challenge with Pseudotyped MERS-CoV

Monoclonal antibodies can provide robust strategy protection against infection caused by MERS-CoV [18,19,39]. In vitro pseudovirus neutralization assays are useful tools to test the efficacy of mAbs [2]. Therefore, the in vitro efficacy of the humanized neutralizing mAb H111-1 to protect R26-hDPP4 mice against infection by pseudotyped MERS-CoV was assessed. As a comparison, the in vitro efficacy of the fully human neutralizing mAb m336 [25] to protect R26-hDPP4 mice against infection was also determined. The 50% inhibitory concentrations (IC_50_s) of mAbs H111-1 and m336 were determined to be 4.5 and 2.7 ng/mL, respectively, representing high in vitro inhibitory activity.

**Figure 6:**
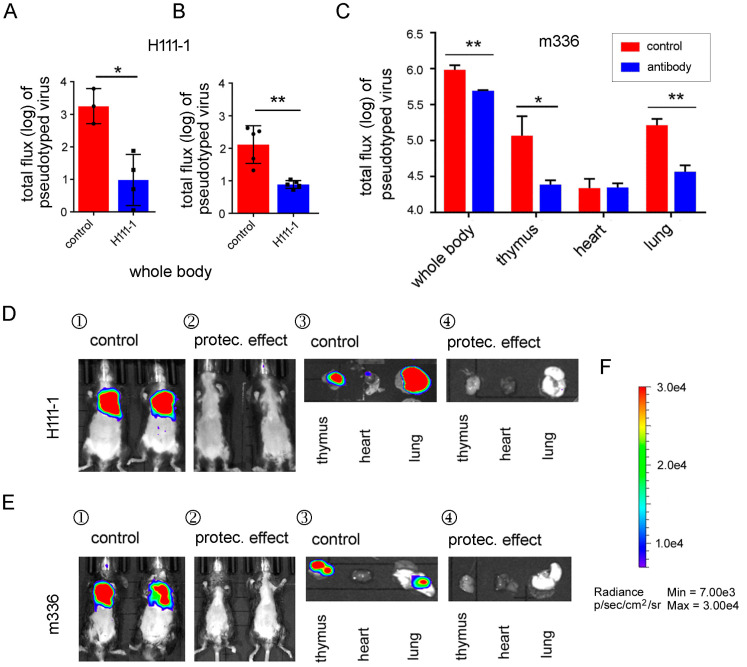
Inhibition of pseudotyped MERS-CoV infection in R26-hDPP4 mice by the novel mAb H111-1 and the well-characterized mAb m336. For evaluation of H111-1, mice were administered 1 mg/kg of mAb either I.P. (**A**) or I.T. (**B**) and 6 h later, challenged with pseudovirus I.T. at a dose of 3.8 × 10^6.5^ TCID_50_. On day 11 p.i., BLI of the whole body or specific organs was conducted (**D**). To evaluate the efficacy of m336 (**C**), mice were administered mAb and challenged using the same doses as for H111-1, and typical images (**E**) are shown. (**F**) Bar of photo flux; for details see Figure 2. *N* = 4 mice in each group, * means *p* < 0.05, ** means *p* < 0.01.

Both mAbs were administered I.T.to R26-hDPP4 mice at a dose of 1 mg/mouse prior to challenge with pseudovirus (1.27 × 10^7.5^ TCID_50_). On day 8 and day 11 p.i., when peak signal could be detected, BLI images of the whole body and organs were obtained. Both mAbs demonstrated strong protective efficacy as shown in Figure 6A–F; H111-1 ablated pseudovirus infection when administered by either the I.P. or I.T. routes (Figure 6A,B,D). The pseudovirus reporter signal in mice administered m336 also decreased significantly in the whole body as well as in lung and thymus (Figure 6C,E,F, *p* < 0.05 or 0.01).

Next, a dose conversion between pseudovirus and authentic virus was calculated in order to establish a safety profile. Three groups of R26-hDPP4 mice were challenged with different doses of pseudovirus (1.25, 3.20, and 3.85 × 10^7^ TCID_50_) either with or without treatment with mAb H111-1 (1 mg/kg) 6 h prior to challenge. BLI of all mice was performed on day 6 p.i. and inhibition rates were calculated. As shown in Figure 4E, when mice were challenged with 1.5 × 10^5^ PFUs of authentic virus, the inhibition rate of H111-1 (1 mg/kg) was 60%. From the inhibition rate curve for pseudovirus infection, we deduced that the pseudovirus dose corresponding to 60% inhibition was 3.25 × 10^7^ TCID_50_. That is, 1 TCID_50_ of pseudovirus was equivalent to 0.0046 PFU of authentic virus. We assumed that this quantitative parameter would be useful for screening antiviral agents and evaluating vaccines against MERS-CoV. Since the R26-hDPP4 mouse provided a suitable model for infection by both pseudotyped and authentic MERS-CoV, the pseudovirus model is a convenient tool to evaluate the in vivo efficacy of anti-MERS-CoV agents or vaccines in most biological laboratories that lack BSL-3 facilities.

### 3.6. A MERS-CoV S-HR1-Specific Fusion Inhibitor Peptide Protected R26-DPP4 Mice from Infection by Pseudotyped MERS-CoV

**Figure 7:**
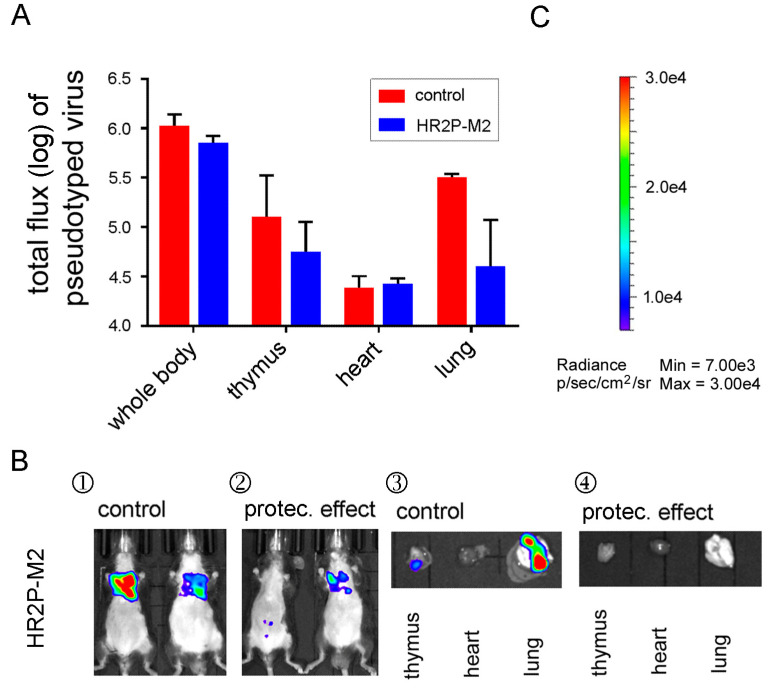
Inhibition of pseudotyped MERS-CoV infection in R26-hDPP4-knockin mice by peptide HR2P-M2. Mice were administered HR2P-M2 peptide or phosphate-buffered saline (PBS) I.T., respectively. Thirty min later, mice were infected I.T. with pseudotyped MERS-CoV (3.8 × 10^6.5^ TCID_50_). BLI images were taken on day 11 p.i., and pseudovirus signals were recorded for the whole body or specific organs. (**A**) Flux value of pseudovirus for assessment of the protective efficacy of HR2P-M2. (**B**) BLI images of whole mice or their organs. (**C**) Bar of photo flux; for details see Figure 2. Four mice were used for each group, and representative images are shown.

Since peptides derived from the heptad repeat (HR)2 of the MERS-CoV S protein such as HR2P or its analogue HR2P-M2 [27] have been shown to protect RAG^−/−^ mice from infection by MERS-CoV (EMC/2012 strain) [26], we investigated whether HR2P-M2 could also prevent pseudovirus infection in R26-hDPP4 mice. We tested the in vitro inhibitory effect of HR2P-M2 in the Huh7 cell line, and its IC_50_ was determined to be 4504.5 ng/mL. In order to determine the in vivo protective effect of HR2P-M2, 5-week-old R26-DPP4 mice were administered 1000 μg of peptide per mouse I.T., and 30 min later challenged with 3.8 × 10^6.5^ TCID_50_ of pseudovirus using the same route. As shown in Figure 7, pseudovirus infection was clearly prevented by HR2P-M2: in one representative mouse, the pseudovirus signal decreased to levels similar to uninfected mice, representing full protection (Figure 7A,B). No or very weak pseudovirus signals were detected in lung, thymus and heart after dissection (Figure 7B), indicating that the R26-hDPP4 mouse model, like previous animal models, can be used for evaluation of antiviral peptides.

## 4. Discussion

MERS-CoV infection causes acute respiratory distress [8,37], neurologic syndromes [40], and death. MAbs are promising tools for both therapeutic and prophylactic interventions against this pathogen [19,41,42,43]. However, the dearth of clinically-effective vaccines and therapeutics compelled us to develop a novel animal model that could faithfully mimic the characteristics of human disease and enable better evaluation of potential vaccines and therapies.

Respiratory disease (such as ARDS) and acute renal failure are the main symptoms of MERS-CoV infection, although patients with fatal disease in the CNS have been reported [40]. Therefore, an animal model of MERS-CoV disease that recapitulates the pathological characteristics in both respiratory tract and CNS, supports high-level virus replication in vivo, and exhibits lung pathology associated with ARDS is required. Moreover, the animal model must be economical, genetically stable, and show reproducible results [8]. Mouse-adapted viral strains have been widely used to establish infection models. Wherever possible, clinical isolates should be used in challenge experiments (http://www.gryphonscientific.com/gain-of-function/) [12]. Targeting transgenes to specific sites of the mouse genome has many advantages over the random insertion typically used in transgenic methods. Two hDPP4-KI mice created by CRISPR/Cas9 gene editing were recently reported [8,12]. In these mice, mDPP4 gene was either replaced by hDPP4 or mutated and its function was disrupted. Since mDPP4 is endowed with different biological activities [44] in immunity and glycometabolism compared with hDPP4, we established KI mice by inserting full-length human DPP4 into the Rosa26 locus via CRISR/Cas9 to spare the function of mDPP4. Notably, the R26-hDPP4 mouse displayed an unusual expression profile, in which transgenic hDPP4 was stably and highly expressed in lung and also expressed to a lesser extent in brain and other organs (Figure 1D–F). We reasoned that this expression pattern might support robust infection and lesions in lung and brain, both using pseudotyped and authentic MERS-CoV. We further reasoned that insertion ofhDPP4 into the safe harbor of the Rosa26 locus and conserving mDPP4 function would be an appropriate strategy for establishing a genetically stable animal model. The insertion of hDPP4 mean, and whether it has implications for the expression of mDPP4 and its biological activities, is not clear.

We observed that R26-hDPP4 mice could be infected by the clinically authentic strain hCoV-EMC [34] at a dose of 1.5 × 10^5^ PFUs, and that infection caused disease symptoms in both lung and brain similar to those observed in clinical patients. Infected R26-hDPP4 mice experienced significant loss of body weight, up to 33.6%, which was comparable to that observed in mice infected with a mouse-adapted strain [21]. Higher viral titers than in previous reports [8] were detected in lungs and were accompanied by diffuse pathogenesis including widened alveolar septa, vascular dilatation and hyperemia, perivascular infiltration of inflammatory cells, necrosis of bronchial epithelial cells, edema and hyaline membrane formation (Figure 2, Supplementary Figure S1). The pathophysiological findings were closely associated with clinical manifestations [45]. All infected R26-hDPP4mice had uniform disease symptoms with a significant difference observed between KI and wild-type mice, as revealed by quantitative pathological scores (Figure 2).

Very few reports have described the sites of MERS-CoV infection in patients, even though these data are crucial for understanding viral pathogenesis. For both cultural and religious reasons, no tissue samples have been made available [37]. However, studies using several animal models has been helpful in addressing this problem. In rhesus macaques, transient lower respiratory tract infection was accompanied by inflammatory cell infiltration [4]. By contrast, severe interstitial pneumonia within filtration and alveolar edema were observed in infected marmosets [6]. Research using small animal models, such as a mouse transduced with an Ad5 vector expressing hDPP4 [9], a Tg mouse model [11], and KI mice created with CRISPR/Cas9 technology [8,12],provide uncontested evidence that the respiratory system is the main site of MERS-CoV infection, in agreement with most clinical manifestations [37]. In this study, we also showed that the lungs, especially the bronchi, were the primary sites of infection both for pseudotyped and authentic virus (Figure 2, Figure 3, Figure 4 and Figure 5; Table S1).

Whether MERS-CoV is able infect the nervous system remains controversial. Agrawal et al. [11] reported high viral loads in the brain, but no necrosis or inflammatory reactions were observed. In KI mice infected with a mouse-adapted viral strain, no viral replication was detected in brain, even at challenge doses of 5 × 10^6^ PFUs. This animal model was heralded as having the advantage of mimicking the clinical manifestations of ARDS in the absence of CNS complications. However, even though respiratory infection and ARDS are common clinical symptoms of MERS-CoV infection, patients with severe neurological syndrome, including confusion, coma and ataxia as well as focal motor deficit, have been reported [40]. Here, when R26-hDDP4 mice were infected with the clinical strain hCoV-EMC at a dose of 1.5 × 10^5^ PFUs, viral loads were detected using IHC assays (Figure 2) in the cerebellum, cerebral ganglia and cerebrum. In association with these viral loads, perivascular gliosis, an indicator of inflammatory reactions, was observed in these organs in all 12 R26-hDPP4mice irrespective of mAb administration or mAb ablation of inflammation (Figure 3). These phenomena were not observed in any wild-type mice challenged in the same manner. Since virus was mainly detected on the membranes of motor neurons, we reasoned that the dyskinesia observed in patients would also be manifested in mice. Additional studies to assess the possible nerve infection are being conducted, and viral loads in nervous system are considered to be titrated.

Pseudotyped virus, which has been widely used for many pathogens [2,30,46], is a useful, safe, and convenient tool for viral infection studies and therapeutic testing [23]. Pseudovirus cell models are popularly applied since they are relatively easy to develop; however, pseudovirus animal models are not as widely available, even though they have more extensive potential applications. Pseudovirus animal models may be difficult to achieve due to lack of susceptibility and the absence of suitably infectious pseudoviruses. Previously, an inhibition assay based on the pseudovirus cell model was established and used to detect neutralizing antibodies against MERS-CoV [2]. However, these pseudoviruses failed to infect the R26-hDPP4 mouse, which was shown to be particularly susceptible to authentic virus. We presumed that this effect might be caused by low pseudovirus titer. Therefore, the codons of the S protein, backbone plasmid [22], and production conditions were optimized systematically, resulting in an increase in titer of about 1000-fold (Supplementary Figure S2) and finally resulting in the successful infection of R26-hDPP4 mice.

To challenge mice, authentic virus is often administered by the I.N. route [18], which has the advantage of mimicking clinical respiratory tract infection. We tried to infect R26-hDPP4-KI mice with pseudovirus by the I.N. route but this was unsuccessful. This failure might have resulted from the use of less than 50 μL of pseudovirus inoculum for nasal dripping; this dosage may not have been sufficient for a single round of infection. We found that different infection routes might result in different biodistribution. Pseudovirus was detected by BLI in the thymus, liver, spleen, kidney, lung, muscle and intestine of mice infected via the I.P. route. However, for challenge via the I.T. route, pseudovirus was only observed in the thymus, heart and lung. Thus, the route of infection also had some effect on pseudovirus distribution. For example, viral loads might fail to be detected in the lungs of mice with minor I.P. infections. However, the I.T. route resulted in uniform infection patterns in the lungs, and especially in the bronchi (Figure 4 and Figure 5). The pseudovirus distribution pattern (Figure 4) was similar to that of Tg mice infected with authentic virus [11]. Mice as old as 9 weeks could be infected, but younger mice (i.e., those 4 to 5 weeks old) had better susceptibility. The pseudovirus infection caused no pathological lesions in multiple organs, indicating that this model was safe (Figure 4).

To verify the potential of the R26-hDPP4 mouse model for evaluating different antiviral agents against MERS-CoV, we tested a newly generated humanized neutralizing mAb, H111-1, as well as a known MERS-CoV-neutralizing mAb, m336, in our pseudovirus model. As expected, m336 and H111-1 both showed outstanding inhibitory activity in the pseudovirus cell model (IC_50_ = 4.5 ng/mL) and pseudovirus mouse model. Moreover, mAb H111-1 was also effective in protecting R26-hDPP4 mice from authentic virus infection. The protective effects of a well-characterized peptide inhibitor, HR2P-M2 [26], were assessed in the pseudovirus model with results similar to those described above. Overall, this pseudovirus mouse model showed outcomes that were consistent with those of the authentic virus mouse model, and the R26-hDPP4-KI mouse is, therefore, a unique animal model for evaluating the in vivo efficacy of anti-MERS-CoV therapeutics and vaccines in biological laboratories lacking BSL-3 facilities.

Some researchers have advanced skepticism regarding pseudovirus models, stating that infection by pseudovirus could not cause pathological changes. To allay these doubts, a combination analysis was deemed necessary in this study. As shown in Figure 5, the relevance of the pseudotyped and authentic virus models was demonstrated. We showed that the manifestations of infection in the lung, the main target of MERS-CoV infection, were very similar between the authentic and pseudovirus models and that in both cases infection was localized to the epithelial cells of the bronchi. A dose conversion between pseudotyped virus and authentic virus was calculated, indicating that this pseudovirus mouse model is reliable and can be used to evaluate the effects of antiviral agents. However, pseudovirus represents a single-cycle infection and without replication in vivo, it does not cause pathological changes. Authentic MERS-CoV infection model is a better choice when studying disease pathogenesis.

In summary, we have presented a novel KI mouse model (the R26-hDPP4 mouse) characterized by its genetic stability, its susceptibility to infection by both authentic and pseudotyed MERS-CoVs, its clear sites of infection, and its presentation of disease symptoms, both in the respiratory tract and CNS, similar to those of clinical patients. These findings imply that this is a unique model for studying human pathogenesis. The established pseudovirus model has the advantages of strong signals, good repeatability and safety, making it as efficient as the authentic virus model for evaluating antiviral agents and vaccines in a typical biological laboratory.

## Figures and Tables

**Table 1:** Administration of neutralizing mAb H111-1 prevented weight loss in R26-hDPP4 mice infected with MERS-CoV *^#^.

Days p.i.	0	1	2	3	4	5
R26-hDPP4 + PBS	0	1.9 ± 2.2	−14.1 ± 1.9	−18.5 ± 5.1	−24.0 ± 4.5	−28.0 ± 5.6
R26-hDPP4 + 1 mg/kgmAbH111-1	0	−0.5 ± 1.4	−13.0 ± 3.5	−17.0 ± 3.0	−22.9 ± 2.7	−27.5 ± 1.9
R26-hDPP4 + 5 mg/kgmAbH111-1	0	−0.1 ± 2.3	−7.6 ± 4.8	−13.1 ± 3.0	−19.0 ± 3.6	−24.8 ± 3.6

Note: * All R26-hDPP4 mice were challenged with authentic MERS-CoV at a dose of 1.5 × 10^5^ PFUs. ^#^ Four mice per group. Numbers represent percentage weight loss compared with weight prior to infection.

## Supplementary Materials

Supplementary materials can be found at https://www.mdpi.com/1999-4915/10/9/448/s1.
