# Correction: Fan et al. A Human DPP4-Knockin Mouse’s Susceptibility to Infection by Authentic and Pseudotyped MERS-CoV. *Viruses* 2018, *10*, 448

**DOI:** 10.3390/v17030363

**Published:** 2025-03-03

**Authors:** Changfa Fan, Xi Wu, Qiang Liu, Qianqian Li, Susu Liu, Jianjun Lu, Yanwei Yang, Yuan Cao, Weijin Huang, Chunnan Liang, Tianlei Ying, Shibo Jiang, Youchun Wang

**Affiliations:** 1 Division of Animal Model Research, Institute for Laboratory Animal Resources, National Institutes for Food and Drug Control, Beijing 100050, China; fancf@nifdc.org.cn (C.F.); wuxi@nifdc.org.cn (X.W.); liususu@nifdc.org.cn (S.L.); caoyuan0512@163.com (Y.C.); chunnan_liang@nifdc.org.cn (C.L.); 2 Division of HIV/AIDS and Sex-Transmitted Virus Vaccines, National Institutes for Food and Drug Control, Beijing 100050, China; liuqiang@nifdc.org.cn (Q.L.); liqianqian1199@163.com (Q.L.); huangweijin@nifdc.org.cn (W.H.); 3 National Center for Safety Evaluation of Drugs, Institute for Food and Drug Safety Evaluation, National Institutes for Food and Drug Control, A8 Hongda Middle Street, Beijing Economic-Technological Development Area, Beijing 100176, China; lujianjun@nifdc.org.cn (J.L.); yangyanwei@nifdc.org.cn (Y.Y.); 4 Key Laboratory of Medical Molecular Virology of the Ministries of Education and Health, Shanghai Medical College, Fudan University, Shanghai 200032, China; tlying@fudan.edu.cn (T.Y.); shibojiang@fudan.edu.cn (S.J.)

## Error in Figure

In the original publication [1], there was a mistake in Figure 3. MERS-CoV S-RBD-specific humanized neutralizing antibody H111-1 protected R26-hDPP4 mice challenged with authentic MERS-CoV as published. Upon review, we identified imaging assembly errors in Figure 3C-7 of the article. We kindly request that the incorrect images be replaced with the corrected versions. The correction this time only replaced Figure 3C-7, which does not affect the conclusion; thus there is no need to modify the original text.

The corrected Figure 3. MERS-CoV S-RBD-specific humanized neutralizing antibody H111-1 protected R26-hDPP4 mice challenged with authentic MERS-CoV as seen below. The authors state that the scientific conclusions are unaffected. This correction was approved by the Academic Editor. The original publication has also been updated.


